# Supplementary material for: Low rates of mutation in clinical grade human pluripotent stem cells under different culture conditions
Source: Nat Commun. 2020 Mar 23;11:1528. doi: 10.1038/s41467-020-15271-3 (PMC7089967; doi:10.1038/s41467-020-15271-3)
Supplement: Supplementary file 1 — Supplementary Information [file 41467_2020_15271_MOESM1_ESM.pdf]

Low rates of mutation in clinical grade human pluripotent stem cells under different culture conditions

Thompson *et al.*

SUPPLEMENTARY FIGURES & TABLES

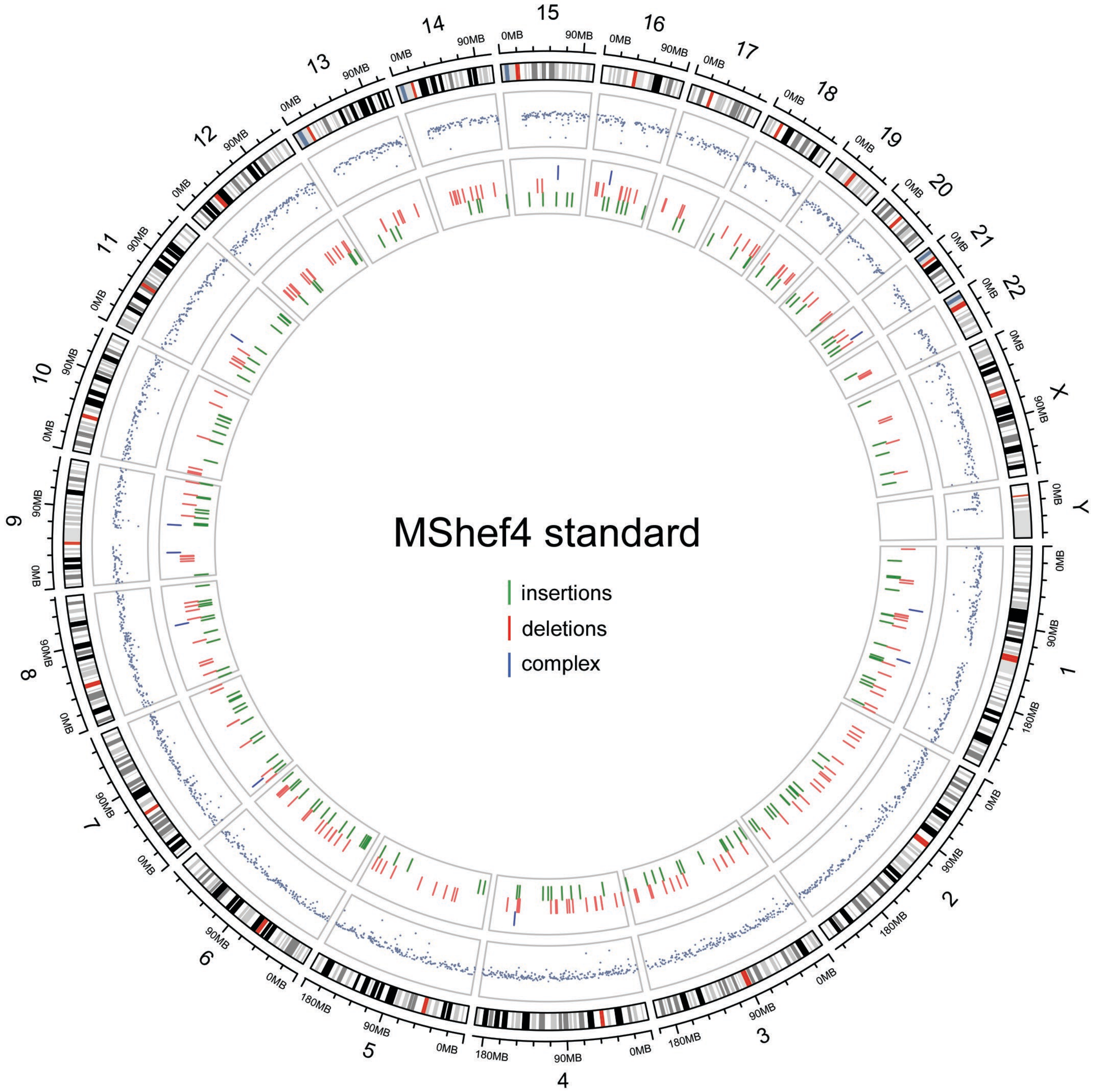

Supplementary Figure 1. Number, location, and class of mutations detected in 20 independant MShef4 subclones grown under standard conditions. Circos plot maps the genomic location of *de novo* SNVs and indels detected in the data: base substitutions (dots; outer track); insertions (inner track; green bars); deletions (inner track; red bars); complex (inner track; blue bars). Source data are provided in Supplementary Data 1.

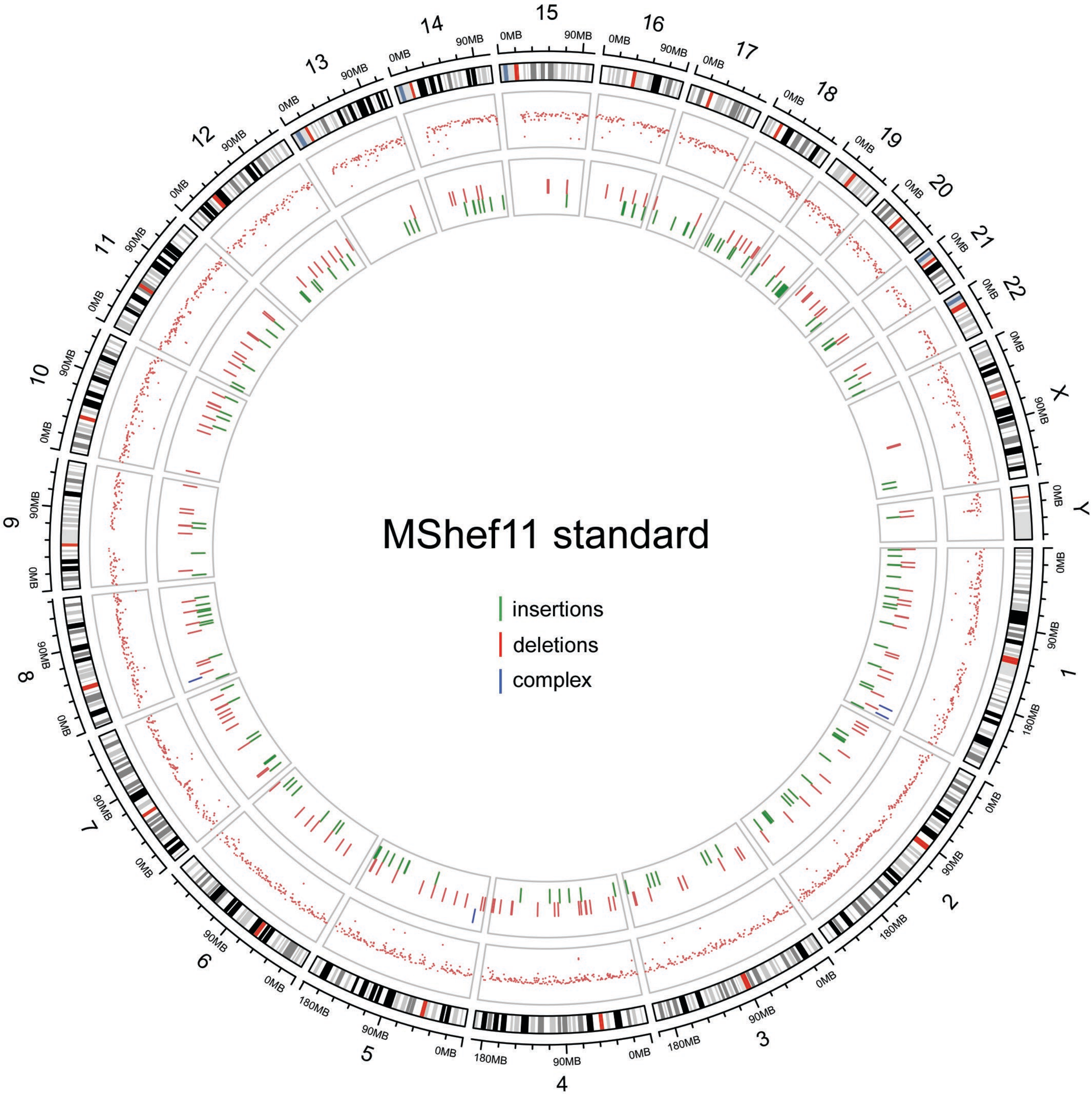

Supplementary Figure 2. Number, location, and class of mutations detected in 19 independant MShef11 subclones grown under standard conditions. Circos plot maps the genomic location of *de novo* SNVs and indels detected in the data: base substitutions (dots; outer track); insertions (Inner track; green bars); deletions (Inner track; red bars); complex (Inner track; blue bars). Source data are provided in Supplementary Data 1.

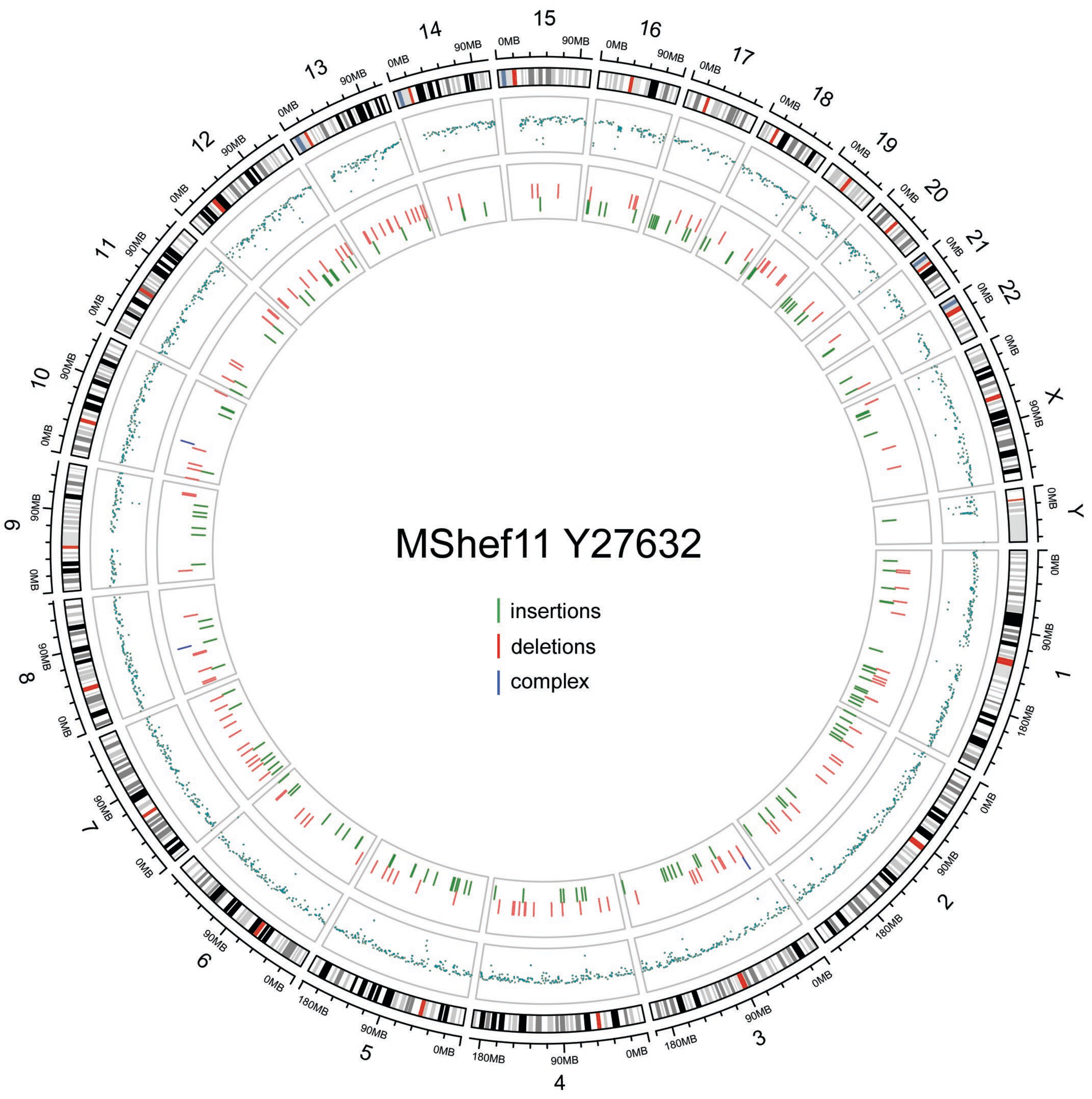

Supplementary Figure 3. Number, location, and class of mutations detected in 20 independant MShef11 subclones grown in the presence of the rho kinase inhibitor, Y27632. Circos plot maps the genomic location of *de novo* SNVs and indels detected in the data: base substitutions (dots; outer track); insertions (Inner track; green bars); deletions (Inner track; red bars); complex (Inner track; blue bars). Source data are provided in Supplementary Data 1.

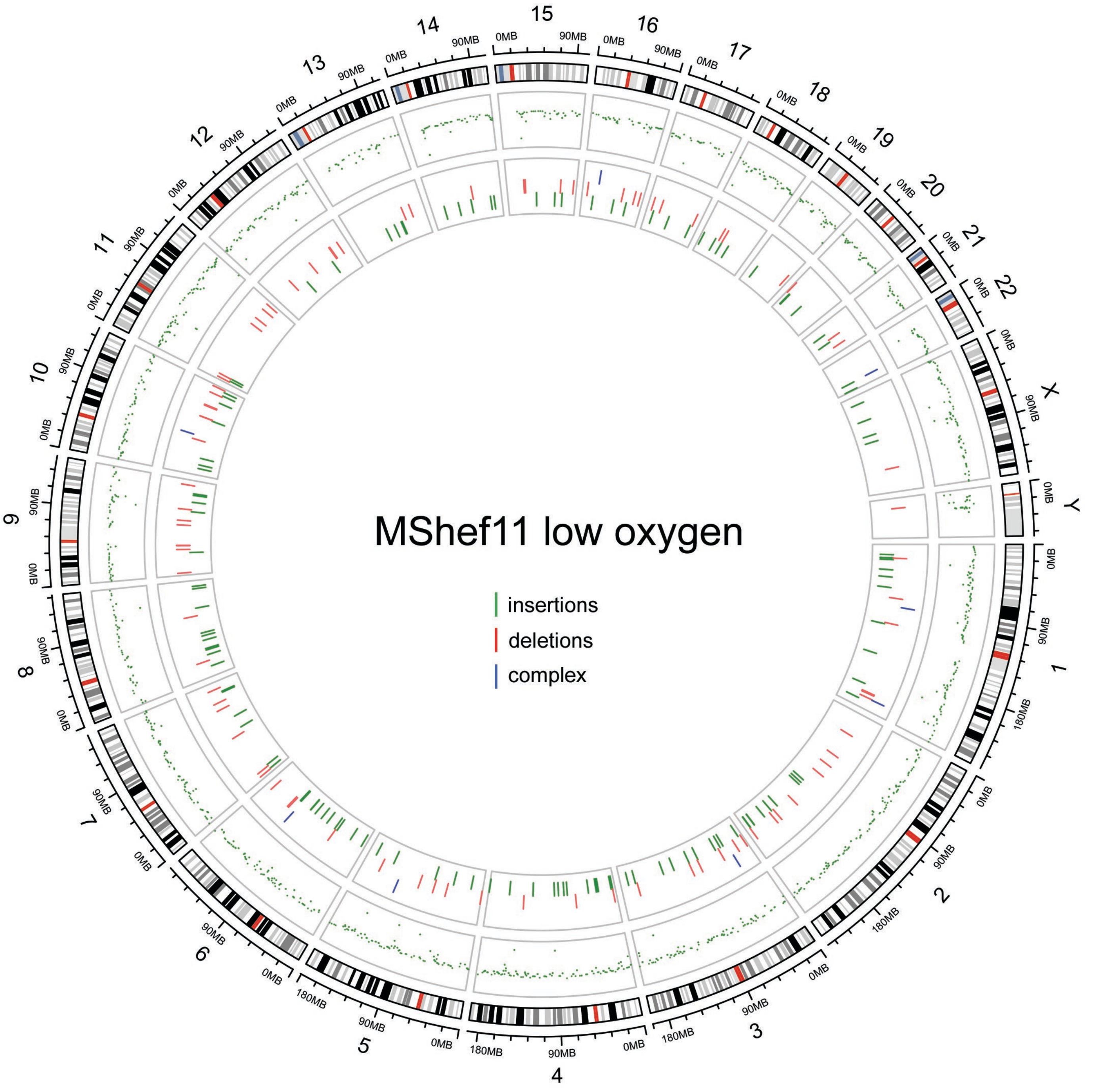

Supplementary Figure 4. Number, location, and class of mutations detected in 21 independant MShef11 subclones grown in low oxygen. Circos plot maps the genomic location of *de novo* SNVs and indels detected in the data: base substitutions (dots; outer track); insertions (Inner track; green bars); deletions (Inner track; red bars); complex (Inner track; blue bars). Source data are provided in Supplementary Data 1.

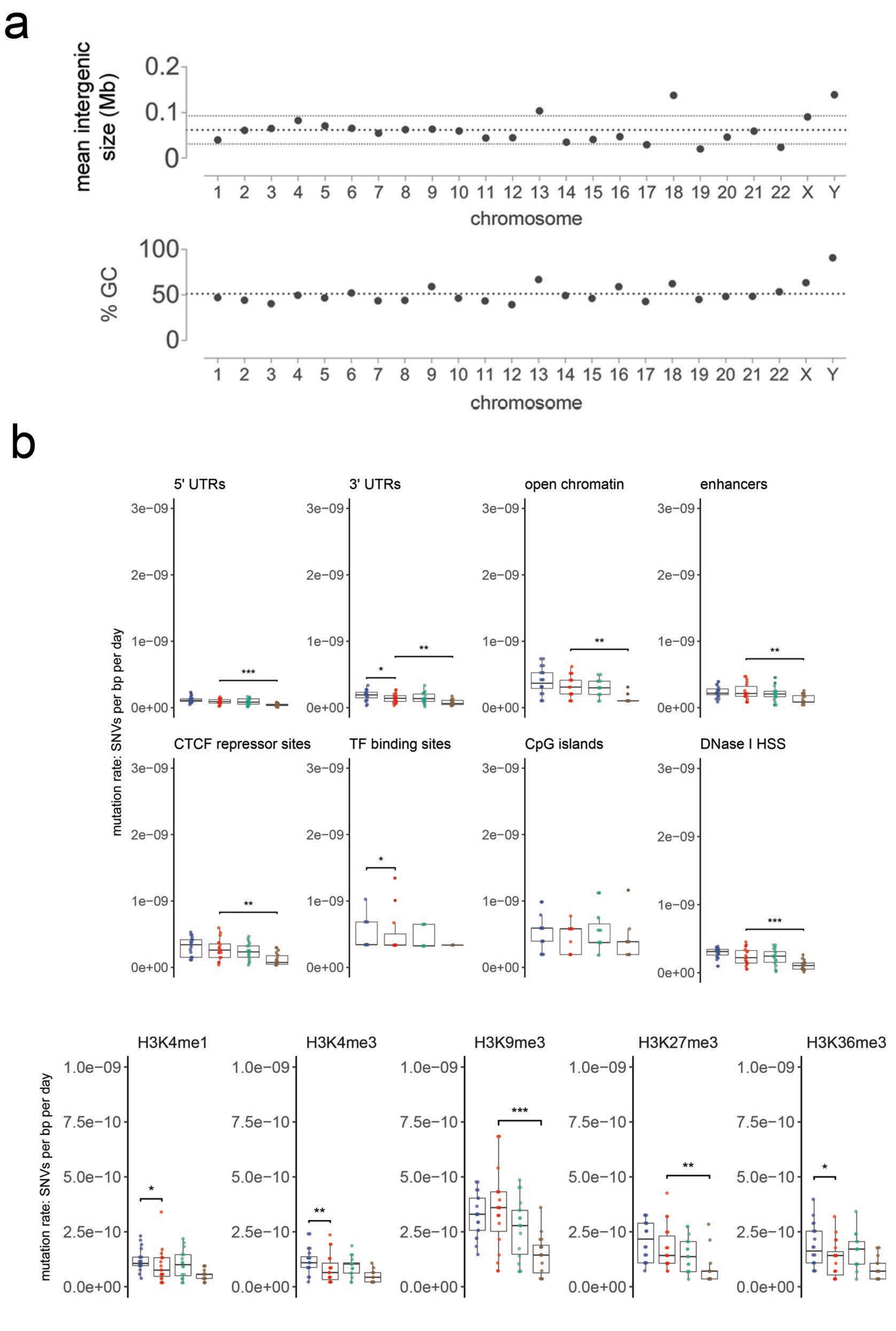

Supplementary Figure 5. Chromosomal GC content and mutation rates across genomic features.
(a) Dot plots indicating the mean intergenic DNA content (top panel) and GC content (bottom panel) of each chromosome, as calculated from the GRCh37 genome assembly. Dashed lines indicates the genome-wide mean for each data set ± SD.
(b) Box and whisker plots showing the mutation rates across a variety of genomic features in MShef4 standard (N = 20), MShef11 standard (N = 19), MShef11 +Y27632 (N = 20), and MShef11 low oxygen (N = 21) subclones. Data show mutation rates normalised to the genomic DNA content of each class of region, as assessed from genome annotation tracks derived from the Ensembl (http://grch37.ensembl.org/index.html) and ENCODE (https://www.encodeproject.org/reference-epigenomes/ENCSR323FKB/) databases. Asterisks indicate level of significance between groups assessed by independent pairwise two-tailed Mann-Whitney tests. (all P-values <0.05). *: P ≤ 0.05; **: P ≤ 0.01, ***: P ≤ 0.001; ****: P ≤ 0.0001. Source data are provided in Supplementary Data 1.

a

b

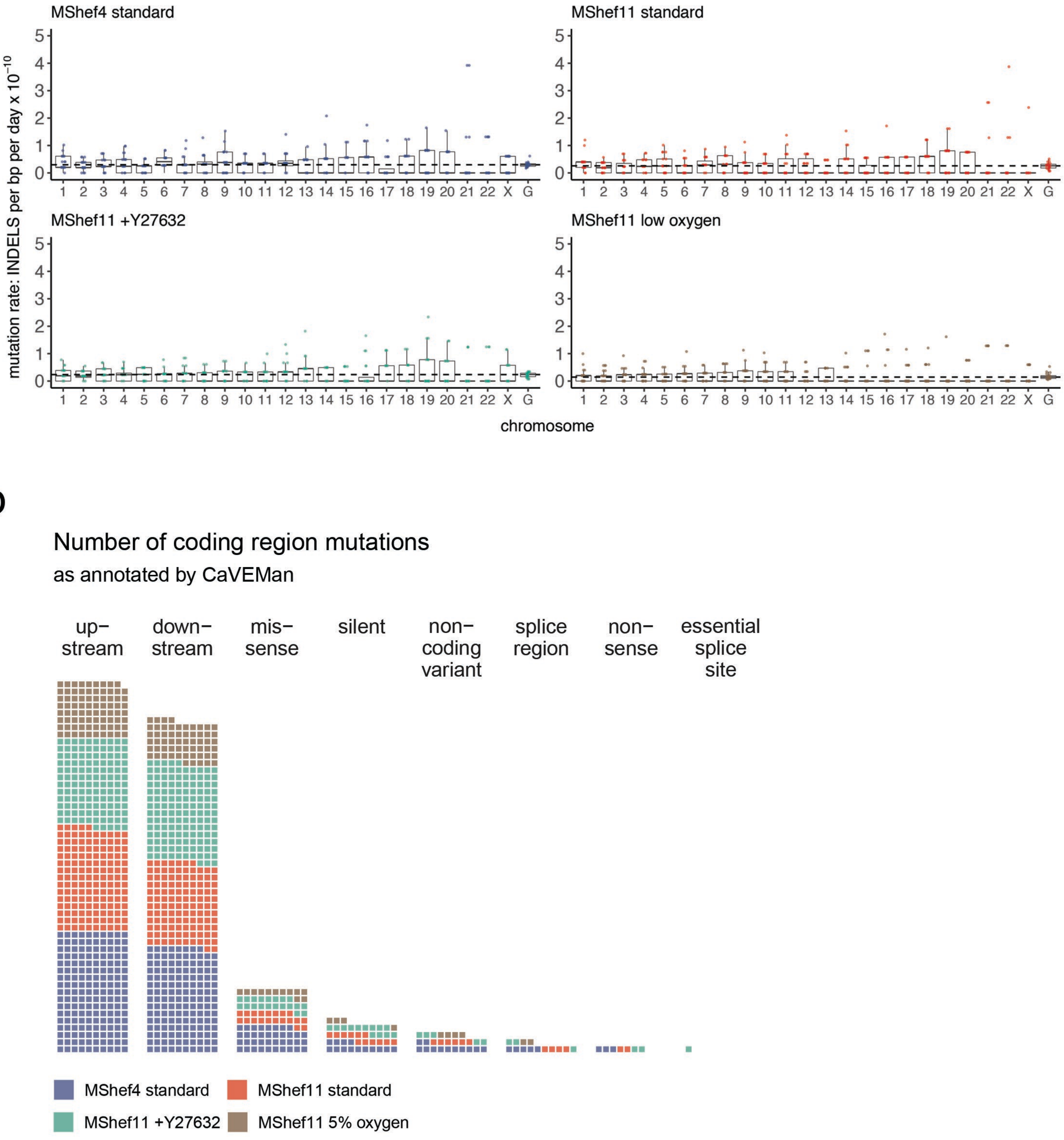

Supplementary Figure 6. Indel mutation rate across chromosomes and mutations detected in coding-regions. **(a)** Box and whisker plots showing INDEL mutation rates of individual chromosomes in MShef4 standard (N = 20), MShef11 standard (N = 19), MShef11 +Y27632 (N = 20), and MShef11 low oxygen (N = 21) subclones grown in different conditions. No systematic deviation from the genome-wide INDEL mutation rate was detected for any individual chromosome. Boxes show 25th-75th percentiles of the data; whiskers show the range of the data; horizontal lines indicate the median value for each group. Asterisks indicate level of significance between groups assessed by independent pairwise two-tailed Mann-Whitney tests. *: $P \leq 0.05$; **: $P \leq 0.01$, ***: $P \leq 0.001$; ****: $P \leq 0.0001$. Source data are provided in Source Data 3.
(b) Waffle chart showing the number of coding-region SNV mutations detected in the data. Source data are provided in Supplementary Data 1

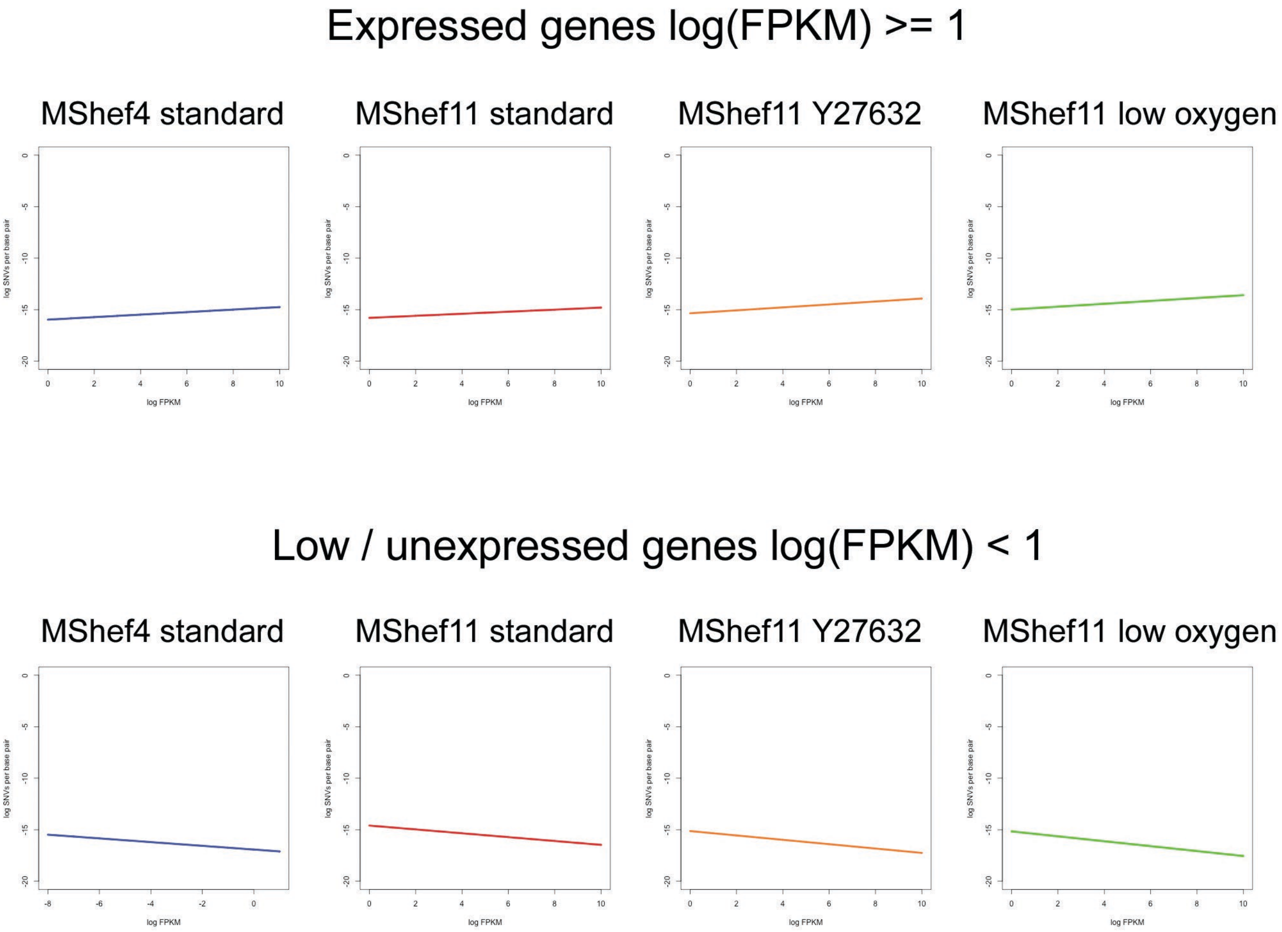

Supplementary Figure 7. Properties of genes with high and low mutational burdens.
For each condition we defined a non-random effect linear model (LMER) to explore the correlation between gene expression and mutation load. The LMER was implemented using the gene length as a non-random factor with the following formula: Mutation_Load ~ Gene_Expression + Mutation_Effect + (1| Gene_Length). In the top panel the LMER is applied to expressed genes (log(FPKM) >= 1) and in bottom panel to low/no-expressed genes (log(FPKM) < 1). Source data are provided in Supplementary Data 5.

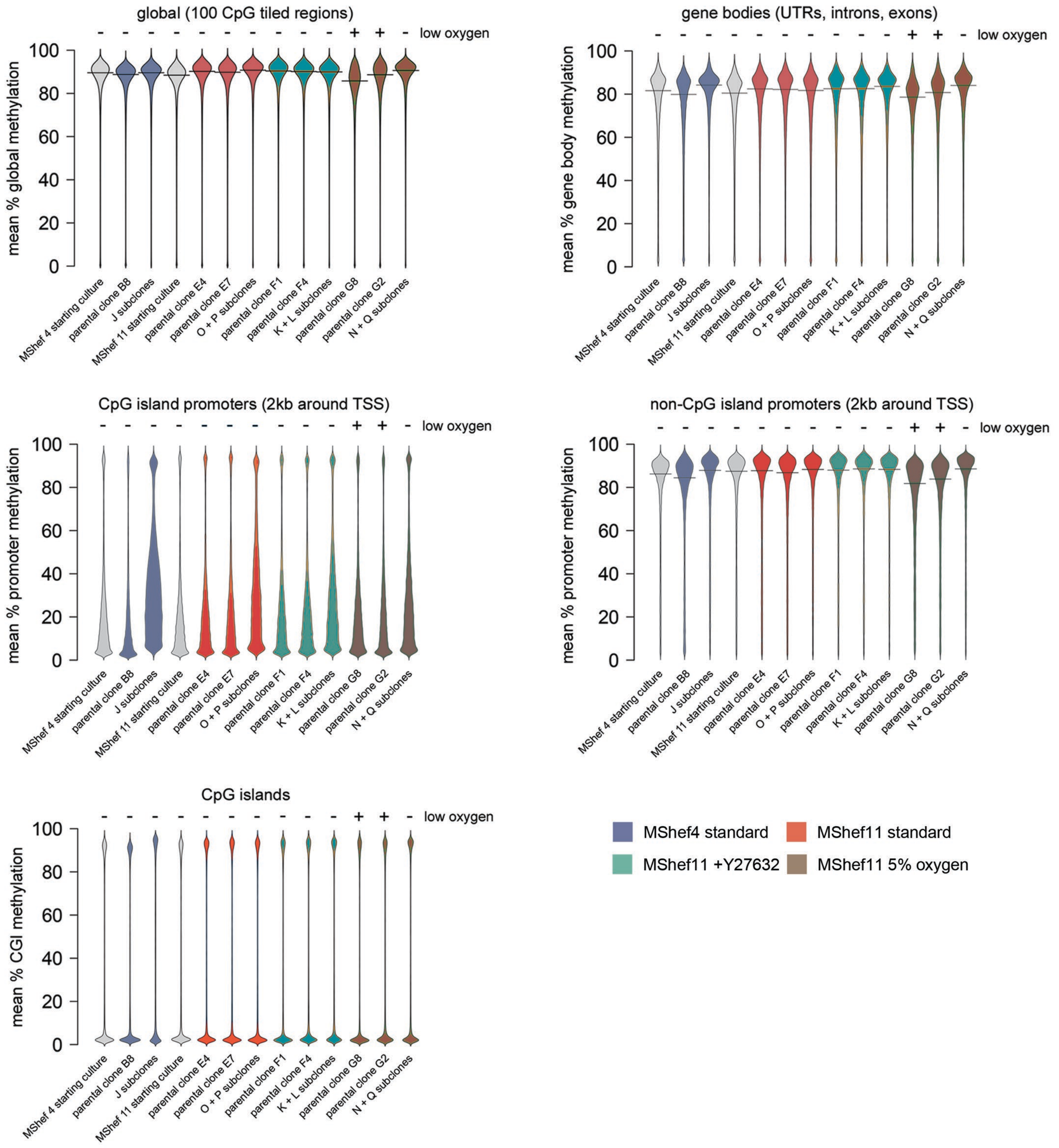

Supplementary Figure 8. DNA methylation levels across the genome.
Beanplots showing DNA methylation levels over different genomic features, across all sample groups. Methylation was assessed globally over tiled genomic regions comprising 100 CpGs, gene bodies (comprising UTRs, introns and exons), CpG islands, and promoters (defined as regions over transcription start sites with 1500kb upstream and 500kb downstream context) with or without CpG islands. Parental clones G2 and G8 expanded in low oxygen show a small decrease in global and gene body methylation compared to other clones and subclones from standard conditions. MShef4 subclones (N = 20) show a large increase in methylation over CpG island-containing promoters compared to their parental clone B8 or the MShef4 starting culture. Source data are provided in Supplementary Data 5-10.

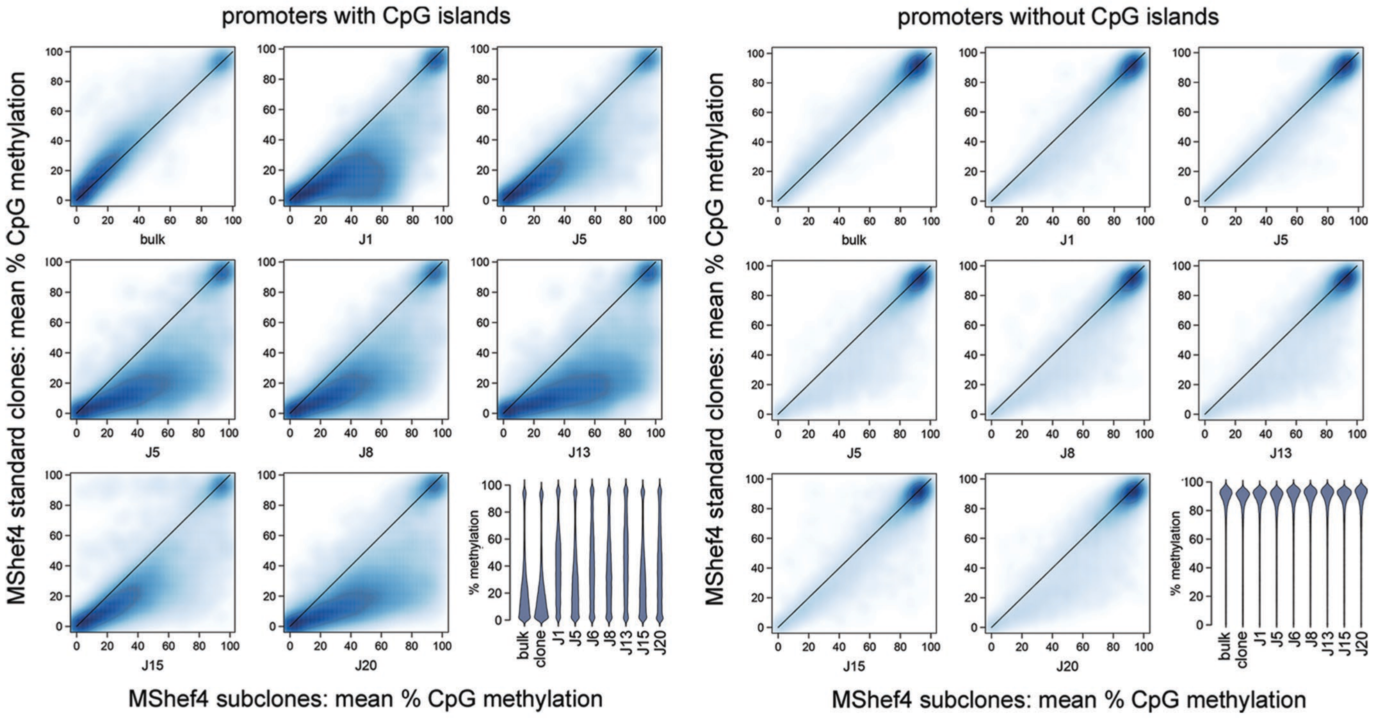

Supplementary Figure 9. DNA methylation levels across promoters.
Density scatter plots and beanplots showing the distribution of CpG island-containing and non-CpG island-containing promoter methylation levels in MShef4 between the starting culture and parental clone B8, and between the parental clone B8 and selected subclones; J1, J5, J6, J8, J13, J15, and J20. Each subclone shows elevated methylation specifically over CpG island-containing promoters. Source data are provided in Supplementary Data 11.

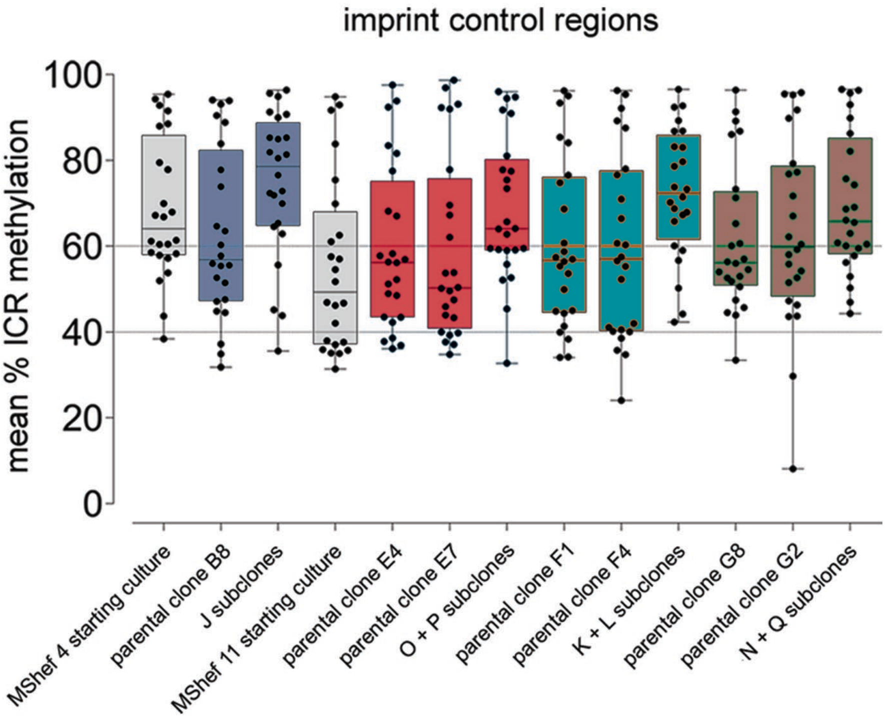

Supplementary Figure 10. Methylation levels of 24 imprint control regions (ICRs) in all sample groups. Box and whisker plots showing the methylation levels of ICRs in the MShef4 starting culture shows high ICR methylation compared to its MShef11 starting culture counterpart. Parental clones B8, E4, E7, F1, F4, G2, and G8 derived from their respective starting cultures do not show significant differences in median methylation, whereas subclone groups (MShef4 standard: N = 20; MShef11 standard: N = 19; MShef11 +Y27632: N = 20; MShef11 low oxygen: N = 21) show elevated methylation compared to parental clones from which they were derived. Dots indicate individual ICRs; boxes represent the 25th-75th percentiles of the data; whiskers show the min and max range of the data; horizontal lines indicate the median methylation level of each group; dashed lines indicate the 40-60% range of methylation. Source data are provided in Supplementary Data 13.

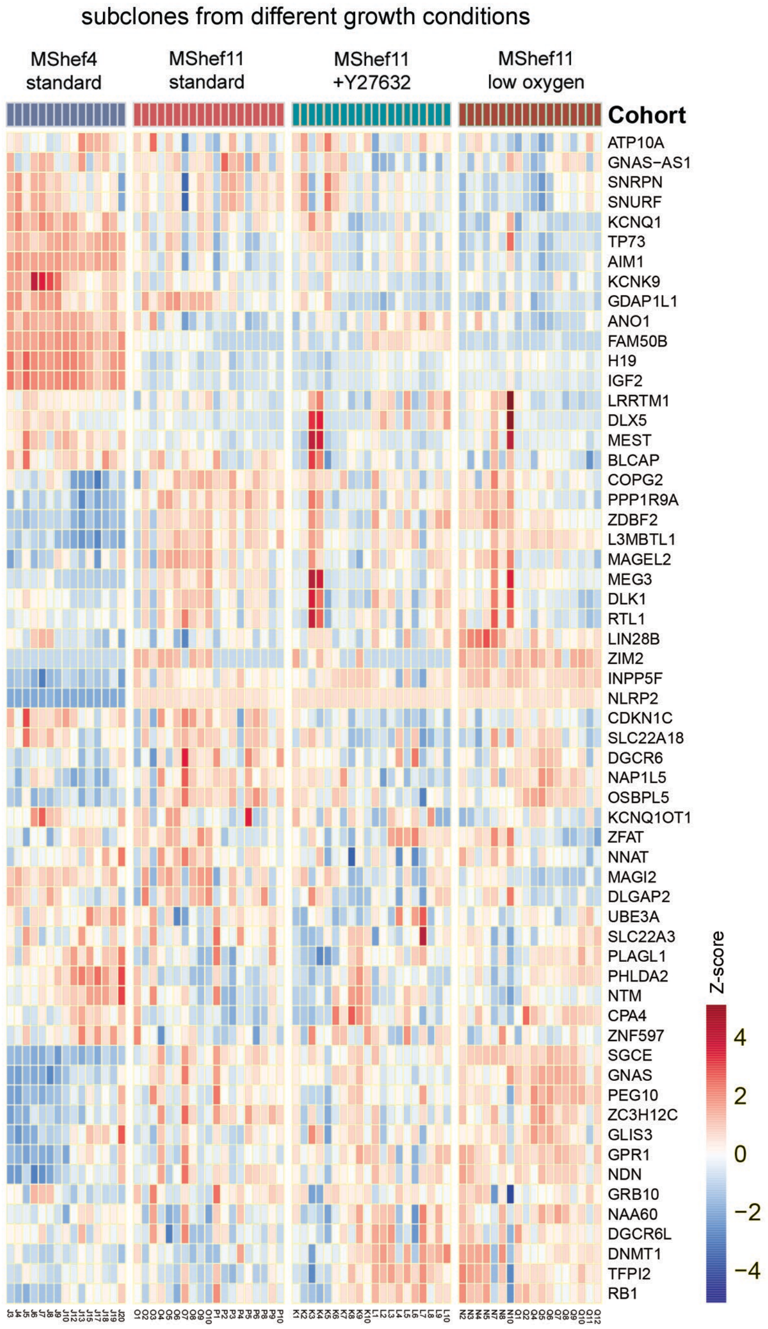

Supplementary Figure 11. Heatmap showing imprinted gene expression in subclones. Heatmap showing the relative expression of 59 imprinted genes, between 15 MShef4 and 57 MShef11 subclones, derived from different growth conditions. MShef11 subclones show a similar pattern of imprint expression between groups, with some exceptions, whilst MShef4 shows a very different pattern of imprint expression to any of the MShef11 groups. Source data are provided in Supplementary Data 13.

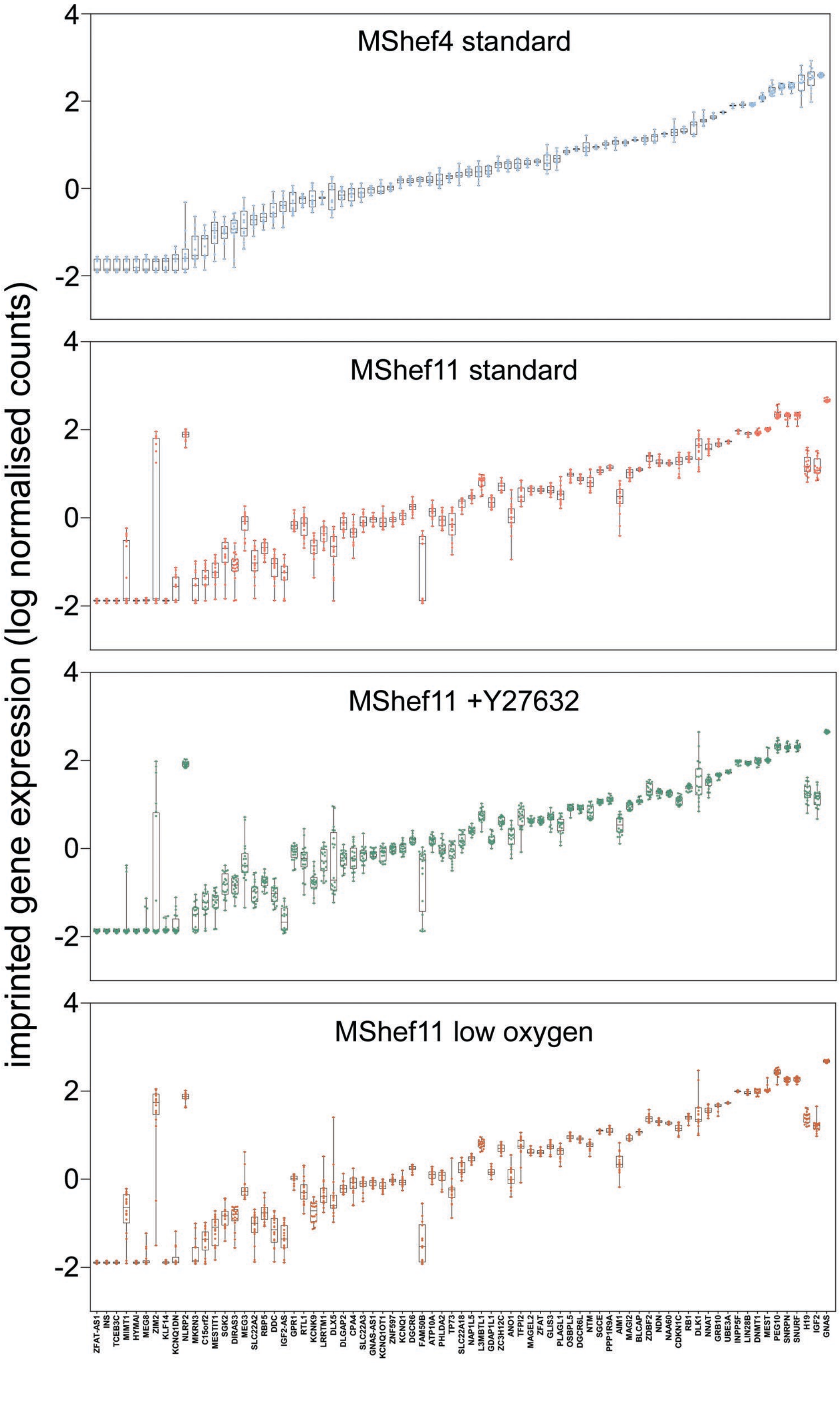

Supplementary Figure 12. Imprinted gene expression in subclones.

Box and whisker plots showing the expression of imprinted genes in MShef4 (N = 19) and MShef11 (N = 57) subclones derived from different growth-conditions. Imprinted genes are denoted along the x axis; log transformed normalised expression counts (fragments per million reads) are shown on the y axis. MShef4 subclones show little variability in imprint expression, whereas MShef11 subclones in each growth condition show large variation of expression for several imprinted genes, and a striking difference in the median expression of some imprints compared to the MShef4 group. For each growth-condition coloured dots indicate imprint expression in individual subclones; boxes represent the 25th-75th percentiles of the data; whiskers show the min and max range of the data. Source data are provided in Supplementary Data 13.

Supplementary Table 1.
Structural Variants detected by the BRASS Algorithm (github.com/cancerit/BRASS) in subclones of MShef4 and MShef11.
Source data are available in Supplementary Data 1.

Subclone	Type	Chr Variant	Break Point 1	Break Point 2	Brass Score
mshef4.J9*	deletion	del(12)(q22q22)	12: 95005259-95005264	12:95390728-95390733	15
mshef4.J19*	deletion	del(10)(q26.13q26.13)	10:121382689-121382742	10:121384139-121384192	19
mShef11.P6*	deletion	del(2)(p16.1p16.1)	2:57770576-57770580	2:57772084-57772088	22
mShef11.O10*	tandem-duplication	dup(4)(q22.2q22.2)	4:93805928-93805938	4:93809164-93809174	27
mShef11.Q9#	tandem-duplication	dup(12)(q14.3q14.3)	12:65016106-65016119	12:65018712-65018725	23
mShef11.P9*	translocation	?der(12)t(8;12)(q21.11;p11.22)	8:73786064-73786067	12:28851692-28851695	25
mShef11.P10*	translocation	?der(12)t(8;12)(q21.11;p11.22)	8:73786064-73786067	12:28851692-28851695	15
mShef11.L2§	translocation	?der(20)t(11;20)(q21;p11.23)	11:93160840-93160841	20:18873936-18873937	19

Notes:
* Normal culture conditions; # Low oxygen culture; § ROCK inhibitor culture
The occurrence of the same translocation, t(8;12)(q21.11;p11.22), in two subclones (P9 and P10) of the same cohort could indicate that this change occurred early in the growth of the parent clone, E7. The sequence data relates genome build GRCh37.

Supplementary Table 2.

Gene expression levels (FPKM) in subclones with nonsense and splice region mutations.

Source data are available in Supplementary Data 4.

Subclone	Mutation Type	Gene	Expression (mutated subclone)	Expression (mean of cohort)	Expression (SD of cohort)	Subclones (N)
MShef4.J13*	Nonsense	*C11orf73*	8.408	12.390	0.692	15
MShef11 K2§	Splice region	*DNAJC6*	2.715	4.935	0.875	20
MShef4 J3*	Nonsense	*SNX27*	19	17	1.58	15
MShef4 J11*	Nonsense	*REV1*	no sequencing data	7.84	0.73	15
MShef4 J16*	Nonsense	*KDM6A*	no sequencing data	18.1	2.16	15
MShef11 O4*	Nonsense	*PSMG1*	26.1	26.9	1	20
MShef11 P5*	Nonsense	*GIMAP8*	0	0	0	20
MShef11 L7§	Nonsense	*LRRC4B*	29.8	36.2	1.58	20
MShef4 J2*	Splice region	*PILRB*	no sequencing data	4.53	1.29	15
MShef4 J5*	Splice region	*KLHL1*	0	0.015	0.02	15
MShef4 J10*	Splice region	*SDSL*	1.67	1.3	0.398	15
MShef4 J15*	Splice region	*RHBG*	0.415	0.829	0.173	15
MShef11 O3*	Splice region	*DAP*	49	53.3	7.85	20
MShef11 O3*	Splice region	*COL22A1*	2.15	2.83	0.351	20
MShef11 O9*	Splice region	*TBC1D9*	3.08	4.53	1.74	20
MShef11 O10*	Splice region	*BFSP1*	1.95	2.03	0.379	20
MShef11 K8§	Splice region	*ITGAX*	0.074	0.15	0.09	20
MShef11 L2§	Splice region	*ESYT2*	18.7	18.5	1.29	20
MShef11 L10§	Splice region	*TECRL*	0.09	0.09	0	20
MShef11 Q3†	Splice region	*EDEM2*	no sequencing data	11.7	0.71	18
MShef11 Q9†	Splice region	*TTN*	0.02	0.03	0.015	18

Notes:

Subclones deviating from the cohort mean are highlighted in grey.

* Normal culture conditions; § ROCK inhibitor culture; † Low oxygen culture.

The means and standard deviations of gene expression are calculated based from the un-mutated subclones in the cohort.

Supplementary Table 3.

Gene enrichment analysis – PANTHER pathway enrichment classification.

Source data are available in Supplementary Data 4

Long genes – low mutation rate

PANTHER Pathways	Number of pathway targets in the Homo sapiens reference genome	Number of pathway targets in the current list (actual number)	Number of pathway targets in a random list of similar size (expected number)	Enrichment (over/under)	Fold Enrichment	Enrichment Raw P-value
Synaptic vesicle trafficking (P05734)	28	3	0.32	+	9.3	5.19E-03
FAS signaling pathway (P00020)	33	3	0.38	+	7.89	7.91E-03
Alpha adrenergic receptor signaling pathway (P00002)	25	2	0.29	+	6.94	3.76E-02
Axon guidance mediated by Slit/Robo (P00008)	26	2	0.3	+	6.67	4.02E-02
Cadherin signaling pathway (P00012)	158	12	1.82	+	6.59	6.10E-07
Alzheimer disease-amyloid secretase pathway (P00003)	69	5	0.8	+	6.29	1.57E-03
Oxytocin receptor mediated signaling pathway (P04391)	58	3	0.67	+	4.49	3.24E-02
5HT2 type receptor mediated signaling pathway (P04374)	67	3	0.77	+	3.88	4.58E-02
EGF receptor signaling pathway (P00018)	134	6	1.54	+	3.88	5.43E-03
Metabotropic glutamate receptor group III pathway (P00039)	68	3	0.78	+	3.83	4.74E-02
FGF signaling pathway (P00021)	120	5	1.38	+	3.62	1.43E-02
Heterotrimeric G-protein signaling pathway-Gq alpha and Go alpha mediated pathway (P00027)	127	5	1.46	+	3.42	1.77E-02
Wnt signaling pathway (P00057)	312	12	3.6	+	3.34	3.64E-04
CCKR signaling map (P06959)	174	6	2.01	+	2.99	1.73E-02
Unclassified (UNCLASSIFIED)	18383	191	211.88	-	0.9	1.80E-04

Short genes – high mutation rate

PANTHER Pathways	Number of pathway targets in the Homo sapiens reference genome	Number of pathway targets in the current list (actual number)	Number of pathway targets in a random list of similar size (expected number)	Enrichment (over/under)	Fold Enrichment	Enrichment Raw P-value
Tetrahydrofolate biosynthesis (P02742)	5	2	0.09	+	23.2	5.69E-03
Formyltetrahydroformate biosynthesis (P02743)	8	2	0.14	+	14.5	1.18E-02
Fructose galactose metabolism (P02744)	12	2	0.21	+	9.67	2.28E-02

Supplementary Table 4: Genotyping of parent clones prior to selection for mutation analysis

**Cell Line**	**Clone[1]**	**Culture Condition[1]**	**Gains of chr 1p, 12p, 17 and 20q detected by FISH[2,3]**	**G-banding Karyotype[2, 4]**	**SNP Analysis[5]**
MShef4	B1	Normal	No anomalies	46,XY [10]	No anomalies
MShef4	B2	Normal	No anomalies	46,XY [13] / 47,XY,+mar [1]	No data
MShef4	B3	Normal	No anomalies	46,XY [7] / 47,XY,+mar [1]	No data
MShef4	B4	Normal	No anomalies	46,XY [14]	No data
MShef4	B5	Normal	No anomalies	46,XY [8]	No data
MShef4	B6	Normal	No anomalies	No metaphases	No data
MShef4	B7	Normal	No anomalies	46,XY [8] / 47,XY,+mar [1]	No data
**MShef4**	**B8**	**Normal**	**No anomalies**	**46,XY [7]**	**No data**
MShef4	B9	Normal	No anomalies	46,XY [23]	No data
MShef4	B1	Normal	No anomalies	46,XY [10]	No data
MShef11	E1	Normal	No anomalies	46,XY [20]	No data
MShef11	E2	Normal	12% +chr20q	No metaphases	No data
MShef11	E3	Normal	No anomalies	No metaphases	No data
**MShef11**	**E4**	**Normal**	**No anomalies**	**No metaphases**	**No anomalies**
MShef11	E5	Normal	No anomalies	46,XY [20]	No anomalies
MShef11	E6	Normal	No anomalies	46,XY [17]	No anomalies
**MShef11**	**E7**	**Normal**	**No anomalies**	**46,XY [20]**	**No anomalies**
MShef11	E8	Normal	No anomalies	No metaphases	No data
MShef11	E9	Normal	No anomalies	No metaphases	No data
MShef11	E10	Normal	10% +chr20q	46,XY [20]	No data
**MShef11**	**F1**	**+Y27632**	**No anomalies**	**46,XY [2]**	**No anomalies**
MShef11	F2	+Y27632	No anomalies	No metaphases	No data
MShef11	F3	+Y27632	No anomalies	46,XY [20]	No anomalies
**MShef11**	**F4**	**+Y27632**	**No anomalies**	**46,XY [20]**	**No anomalies**
MShef11	F5	+Y27632	12% +chr20q	No metaphases	No data
MShef11	F6	+Y27632	No anomalies	46,XY [16]	Higher LRR on Ch15
MShef11	F7	+Y27632	No anomalies	No metaphases	No data
MShef11	F8	+Y27632	No anomalies	46,XY [20]	No data
MShef11	F9	+Y27632	No anomalies	46,XY [8]	No data
MShef11	F10	+Y27632	10% +chr20q	47,XY [9]+mar [1]	No data
MShef11	G1	Low O_2	No data	46,XY,del(11p) [1] /46,XY [19]	No data
MShef11	G2	Low O_2	No anomalies	No metaphases	Low LRR
MShef11	G3	Low O_2	No anomalies	46,XY [20]	Low LRR on Chr 11,15
MShef11	G4	Low O_2	No anomalies	46,XY [12]	No data
MShef11	G5	Low O_2	No anomalies	No metaphases	No data
MShef11	G6	Low O_2	No anomalies	46,XY [20]	No data
MShef11	G7	Low O_2	No anomalies	46,XY [20]	No data
**MShef11**	**G8**	**Low O_2**	**No anomalies**	**46,XY [9]**	**No data**
MShef11	G9	Low O_2	No anomalies	No metaphases	Low LRR in Chr11
MShef11	G10	Low O_2	10% +chr20q	No metaphases	No data

Supplementary Table 4 Notes:

1. Clones were derived by single cell deposition and expanded in the relevant growth conditions as described in the Materials and Methods. To select those clones that would be taken forward for analysis, samples of cells from each clone were diverted at the earliest possible time for an initial screen for any substantial genomic change by Fluorescent In Situ Hybridisation (FISH), backed up by G-banding karyotyping and SNP array. Unfortunately, because of the small amount of material available at these early stages, not all of these assays were successful in every case. The clones selected for the mutation rate analysis are highlighted in yellow.

2. Cells harvested for interphase FISH and G-banding karyotype two passages post cloning.

3. We used interphase FISH to screen for cells with gains in chromosomal regions commonly subject to amplification: chromosomes 1q, 12p, 17p, 17q, 20q. In each case, 100 interphase nuclei were scored. The following probes were used:

Chr. 1p36: probe, LSI 1p36 (Abbott, Vysis Cat No. 04N60-020)
Chr. 1q25: probe, LSI 1p25 (CE) (Abbott, Vysis Cat No. 04N60-020)
Chr. 12p11.1-q11(centromere): probe, CEP 12 (Abbott, Vysis Cat No. 07J20-01)
Chr. 17p13: probe, Iso 17q (Leica, Kreatech Cat No. KCN-10011)
Chr. 17q22: probe, Iso 17q (Leica, Kreatech Cat No. KCN-10011)
Chr. 20q11.21: probe, RP5-857M17 (Illumina, BlueGnome)
Chr. 20q13.3 (telomere): probe, TelVysion 20q (Abbott, Vysis Cat No. 05J04-020)

4. For G-banding karyotype, the number of metaphases screened is indicated in [ ]; because of the small numbers of cells available to screen at this early stage, in some cases rather fewer metaphases were analysed than we would normally wish to screen, and in some cases no metaphases were obtained.

5. For SNP analysis, DNA was harvested 3 passages after cloning, although in some cases there was insufficient DNA for analysis. SNP array analysis was conducted using Illumina Human Cyto Omin25M_8v1.1 Bead Arrays. The B allele frequencies (BAF) and the log R ratio (LRR) is the log2-transformed value of the normalised intensity of the SNP bead, (RA + RB)/R-expected, where Rexpected is calculated interpolating of the reference genome. BAF and LRR were calculated for the full genome and compared across samples to detect abnormalities. With No data we refer to clones that have not been screened using the Bead Arrays. With No anomalies we refer to clones that had comparable BAF across the whole genome and LRR within the normal range ($-0.5 \leq LRR \leq 0.5$). Anything detected has been specified in the table in details.
