# Supplementary material for: Low rates of mutation in clinical grade human pluripotent stem cells under different culture conditions
Source: Nat Commun. 2020 Mar 23;11:1528. doi: 10.1038/s41467-020-15271-3 (PMC7089967; doi:10.1038/s41467-020-15271-3)
Supplement: Supplementary file 21 — Description of Additional Supplementary Files [file 41467_2020_15271_MOESM21_ESM.pdf]

Title: Supplementary Data 1:
Description: De novo SNVs identified in MShef4 and MShef11 parent clones and subclones

(relates to Figure 2; Supplementary Figures 1-6; Supplementary Table 1)

Title: Supplementary Data 2:
Description: Mutational signatures identified in MShef4 and MShef11 subclones

(relates to Figure 3)

Title: Supplementary Data 3:
Description: INDELs detected in the MShef4 and MShef11 parental clones and subclones.

(Relates to Figure 4; Supplementary Figure 6)

Title: Supplementary Data 4:
Description: Expression of genes

(Relates to Figure 5 and Supplementary Tables 2 and 3)

Title: Supplementary Data 5:
Description: Genes with high and low mutational burden

(Relates to Figure 5 and Supplementary Figure 7)

Title: Supplementary Data 6:
Description: Global methylation

(Relates to Figure 6; Supplementary Figure 8)

Title: Supplementary Data 7:
Description: Gene methylation

(Relates to Supplementary Figure 8)

Title: Supplementary Data 8:
Description: CpG island methylation

(Relates to Supplementary Figure 8)

Title: Supplementary Data 9:
Description: Promoter methylation

(Relates to Supplementary Figure 8)

Title: Supplementary Data 10:
Description: Methylation of CpG island-containing promoters

(Relates to Figure 6; Supplementary Figure 9)

Title: Supplementary Data 11:
Description: Methylation of non-CpG island-containing promoters

(Relates to Figure 6; Supplementary Figure 9)

Title: Supplementary Data 12:
Description: Hypermethylated CpG island-containing promoters in MShef4 subclones

(Relates to Figure 6)

Title: Supplementary Data 13:
Description: Expression of DNMT genes

(Relates to Figure 6)

Title: Supplementary Data 14:
Description: ICR methylation, imprint expression and miRNA expression

(Relates to Figure 6; Supplementary Figures 10-12)

Title: Supplementary Data 15:
Description: Statistics related to sequencing coverage for WGS (Relates to Methods)

Title: Supplementary Data 16:
Statistics related to sequencing coverage for WGBS (Relates to Methods)

Title: Supplementary Data 17:
Description: Statistics related to sequencing coverage for RNA-seq (Relates to Methods)
